# De-contextual communication: Factors influencing usage intentions of metaverse technology in digital library services

**DOI:** 10.1016/j.heliyon.2023.e20388

**Published:** 2023-09-26

**Authors:** Sri Sediyaningsih, Mohammad Pandu Ristiyono, Kani Launggu, Peter Ochieng Juma

**Affiliations:** a Communication Science Study Program, Faculty of Law, Social and Political Science, Universitas Terbuka, Jl. Cabe Raya, Pondok Cabe, Pamulang, Tangerang, Jakarta, 15418, Indonesia; b Department of Library and Archives, Universitas Terbuka, Jl. Cabe Raya, Pondok Cabe, Pamulang, Tangerang, Jakarta, 15418, Indonesia; c Information System Study Program, Faculty of Mathematics and Natural Science, Universitas Terbuka, Jl. Cabe Raya, Pondok Cabe, Pamulang, Tangerang, Jakarta, 15418, Indonesia; d Institute of Informatics, University of Szeged, Arpad ter, Szeged, H-6720, Hungary

**Keywords:** De-contextual communication, Metaverse technology, Digital library services, Unified system information theory, PLS-SEM

## Abstract

This study aims to investigate the factors influencing the usage intentions of metaverse technology in digital library services within higher learning institutions, using the unified system information theory. To achieve this, an online survey was conducted among university staff and students, utilizing a link-scale measurement. Various factors affecting the usage intention of metaverse technology in library services were computed through transformation models such as UTAUT, DM, ISS, and TTF. Subsequently, the model parameters were empirically tested using partial least squares structural equation modeling (PLS-SEM) to identify the significant factors influencing the usage intention of metaverse technology. The results of the study reveal that users' intentions to use metaverse technology in digital library systems are influenced by perceptions of system use, perceived interaction, perceived usefulness, and perceived ease of use. Notably, these influences vary depending on the user's intended task. These findings provide valuable insights into the factors that affect the adoption and usage intentions of metaverse technology in the context of digital library services in higher learning institutions. This research contributes to enhancing understanding and guiding future strategies for leveraging metaverse technology effectively in educational environments.

## 1 Introduction

The COVID-19 outbreak presented numerous challenges to higher learning institutions, compelling them to explore new digital solutions to ensure continued education. One of the recent technologies introduced during this period is the metaverse library, which leverages teleworking capabilities to minimize the risk of transmission [1].

Distance learning in higher education heavily relies on digital platforms for communication, interaction, and the delivery of learning materials [2]. Consequently, the design of the system and the technology utilized play critical roles in determining user satisfaction, usage intentions, and adaptability to the deployed system, ultimately influencing user behavior towards the technology [3], [4]. Additionally, the diverse nature of distance learning and the heterogeneity among students further contribute to the usability considerations of metaverse technology [5].

The concept of the metaverse has captured the imagination of both the literary world and the technology industry. Coined by Neal Stephenson in his novel “Snow Crash” in 1992, the metaverse refers to a virtual reality world where individuals can engage with others through avatars [6]. While early attempts at creating metaverse-like platforms, such as Second Life, failed to gain significant popularity, recent technological advancements and shifting societal needs have sparked a renewed interest in the potential of the metaverse. The limitations of networks and devices in supporting 3D graphics during the early 2000s hindered the widespread adoption of metaverse platforms [7].

The metaverse's potential is being reevaluated due to various factors such as 5G networks, deep learning technology, virtual reality devices, NFTs, cryptocurrency, and the COVID-19 pandemic's impact on non-face-to-face activities [8]. Notably, significant players in the tech industry have taken notice of the metaverse's potential. In April 2021, Nvidia CEO Jensen Hwang announced the company's intention to develop a metaverse, and in October 2021, Facebook CEO Mark Zuckerberg changed the company's brand name to Meta, emphasizing its commitment to building a metaverse [9].

These developments have prompted a fresh examination of the metaverse's potential for individual users and businesses alike. For individual users, the metaverse offers the ability to seamlessly navigate between the real world and a virtual world limited in social sign. The novel and subsequent film adaptation of “Ready Player One” depict a future where individuals, particularly the younger Generation Z, immerse themselves in a virtual world, finding connection, satisfaction, and novel experiences [5].

Platforms like Geppetto and Roblox have amassed millions of users, especially children and teenagers, who embrace the interconnected identities of the real and virtual worlds [10], [11]. Tech giants have strategically built business models around metaverse platforms, which play crucial roles in connecting the virtual world with the real world. These models enable the creation and exchange of value within the metaverse ecosystem, benefiting both the companies and the users [12]. Meta's launch of the Oculus Quest 2 [13] virtual reality headset and the Horizon virtual world platform, which includes Horizon Workroom Beta for virtual offices, exemplify the application of the metaverse in redefining internet usage, communication, and work [2], [4]. While the metaverse has garnered significant attention, its applications in digital library services remain relatively unexplored [3].

Research into metaverse technology in digital library services is in its infancy, with a limited understanding of the factors that drive user demand and influence continuous usage intention [14]. Existing theoretical models for information system adoption have addressed user behavioral intention, but the challenge lies in ensuring user satisfaction. In the customer-centric era, providing a satisfactory user experience is crucial to fostering loyalty and continuous usage [15].

DeLone and McLean's (DM) [16], Information System Success (ISS) [17], Unified Theory of Acceptance and Use of Technology (UTAUT) [18], and Task Technology Fit (TTF) [19] models offer insights into user satisfaction and continuous usage intention. Combining these models can provide a comprehensive understanding of the factors that influence the sustained usage of metaverse technology in digital library services [15]. Key factors, including system quality, information quality, service quality, collaboration quality, and task-technology fit, contribute to user satisfaction and behavioral intention [15].

This study aims to fill a research gap by investigating the factors influencing users' continuous usage intention of metaverse technology in digital library services. By integrating the DM ISS, UTAUT, and TTF models, the research seeks to identify the key drivers behind sustained usage. Valuable insights from this study will aid service providers in the digital library sector to develop effective strategies for user retention and the long-term development of metaverse technology.

Moreover, this research examines the usage intentions of metaverse technology in library services within a higher learning institution, specifically in the context of higher education. By analyzing user behavior and satisfaction, the study aims to provide valuable insights into enhancing the implementation and acceptance of metaverse technology in educational settings.

The rest of this paper is organized as follows: In section 2, we provide a brief literature review related to our study; In section 3, we define our hypothesis; In section 4, we give a brief description of our methodology; In section 5 we present our results; In section 6, we give summary discussion. Finlay in section 7 we give conclusions and recommendations.

## 2 Literature review

### 2.1 Social presence theory

The concept of social presence theory explores the projection, emotional connection, and sense of being present with others during interactions [20]. Its relevance persists in understanding nonverbal cues and is particularly important in online interactions within distance education [21]. The theory now extends to encompass computer-mediated communication, as new forms of communication continue to evolve [22]. Computer-mediated communication (CMC) encompasses various digital communication methods, such as online social networks, chat rooms, and virtual communities, provides individuals with opportunities to interact, share information, and engage in social relationships. On the other hand, social presence refers to the perception of others' presence in mediated environments [23]. The relationship between CMC and social presence is intricate. CMC tools provide avenues for expression, facilitating social presence through text, images, audio, and video. Though lacking non-verbal cues, CMC still allows for the transmission of visual and auditory cues via emoticons, emojis, and multimedia content [24]. Perceived intimacy, formed through private conversations, enhances social presence [25]. Additionally, CMC enables the formation of online communities, fostering a sense of belonging. Anonymity in CMC may affect social presence, as it can lead to disinhibition but reduce accountability. However, the absence of non-verbal cues in CMC can diminish social presence. It's crucial to consider individual characteristics, context, and technology when examining the association between CMC and social presence.

### 2.2 De-contextual communication

De-contextual communication, also referred to as virtual communication, is a form of interaction that occurs through mediums like computer-mediated communication (CMC). It involves the exchange of information and ideas between two or more individuals using various media platforms [26]. It can be understood as a type of communication that takes place in virtual worlds or through computer-mediated channels. The concept of virtual reality (VR) as a novel communication medium continues to develop as people explore and engage with it [27]. Recent studies have predominantly focused on the social implications of de-contextual communication [28]. Due to the absence of social cues and nonverbal gestures inherent in face-to-face interactions, this form of technology-mediated communication is characterized as de-contextualized [29]. While computer-mediated communication enables individuals to connect across different locations and time zones, it also presents challenges, as certain social cues may be absent from the interaction process. Although face-to-face communication is widely regarded as more effective than computer-mediated communication (CMC) in terms of conveying social signs, recent studies have demonstrated that CMC is actually more effective and popular, particularly when targeting larger audiences [30]. This indicates that computer-mediated communication, despite its ability to facilitate interaction, may fall short of completely replicating the intimacy found in face-to-face communication. Additionally, it has the potential to transcend the boundaries of traditional social spheres [31].

### 2.3 Digital ecosystem

The term “digital ecosystem” refers to a dynamic and interconnected environment where various elements such as human beings, information services, network interactions, knowledge-sharing tools, and resources come together to facilitate collaboration and synergy among individuals or organizations [32], [33]. It can be seen as a community of interconnected digital communities, comprised of different digital species, that operate within a digital realm. These communities interact as functional units, sharing actions and information flows [34], [35]. In essence, a digital ecosystem fosters an inclusive community in a digital space, bringing together individuals, information services, and knowledge-sharing tools to enable the distribution of resources for the benefit of individuals or organizations [36]. Additionally, a digital ecosystem relies on an information infrastructure and a collaborative community that engages with one another, supported by various sources of information and the utilization of electronic devices and networks [37], [38].

### 2.4 Metaverse

Neal Stephenson's novel “Snow Crash” introduced the term “metaverse” as a transformative concept in the world of Web 3.0 [39]. By definition, the metaverse is an immersive mixed-reality world that encompasses virtual realms alongside the physical world or augmented real-world data [40]. This study explores user acceptance of a metaverse technology on a digital library platform, where people can interact, collaborate, and live without limitations of time and space [10]. The metaverse represents a fusion of physical and virtual realms, offering unparalleled opportunities for communication, interaction, and value creation in immersive Internet platforms [41], [42].

The Metaverse offers a transformative platform that simplifies and enhances library services, enabling easier access to information and resources [43]. By leveraging virtual reality, augmented reality, and interconnected digital spaces, the Metaverse revolutionizes the way libraries can serve their patrons [44]. Users can navigate virtual libraries, interact with digital collections, attend immersive educational programs, and engage in collaborative learning experiences seamlessly. The Metaverse empowers libraries to transcend physical boundaries, fostering inclusivity and providing a dynamic and accessible environment for individuals to explore, learn, and connect with knowledge in a more convenient and engaging manner [45].

The metaverse, with its vast potential and importance, is currently lacking in dedicated research. Existing studies can be categorized into two types: those that define and conceptualize the metaverse, and those that examine its impact on various aspects of our lives and business [46]. These studies explore key elements such as avatars, virtual worlds, interaction, immersion, social collaboration, and permanence. However, a comprehensive understanding of the metaverse and its implications is yet to be achieved [47].

### 2.5 DeLone and McLean's Information System Success (DM ISS)

This study utilized DeLone and McLean's Information System Success (DM ISS) framework [48] to examine the impact of satisfaction and performance expectations on the intention to use mobile payment systems. The integration of DM ISS into UTAUT allowed for the measurement of the information system. User satisfaction and performance expectations were found to be influenced by system quality (SYQ), service quality (SQ), and information quality (IQ), which ultimately affected the intention to use. The ISS has been widely employed to assess users' readiness to adopt various information systems following its redefinition.

In a related study by [49] integrated DM Information System Success (ISS) into the Technology Acceptance Model (TAM) to evaluate the influence of effective facilitators on user satisfaction, performance expectations, and the usability of e-learning [50]. A similar study by [51] expanded the DM ISS to investigate user intention towards digital library services and proposed a revised conceptual framework. These investigations shed light on the use of DM ISS in understanding user behavior and intention to use different systems. However, the application of DM ISS in the field of digital library services, which represents cutting-edge educational technology, has been largely unexplored. Therefore, it is essential to apply the DM ISS model as a conceptual framework for metaverse digital library services [52]. By doing so, researchers can gain insights into user behavior and intention toward this innovative educational technology.

### 2.6 Task Technology Fit (TTF)

The concept of Task Technology Fit (TTF) evaluates how well technical capabilities meet the requirements of a specific task. According to this theory, the intention to use technology and the expectation of its performance is influenced by the technology's ability to adapt its functionality to the needs of the mission [53]. When the technical features are well-suited and adjusted, it is encouraged to employ digital library technology. Conversely, if the technical features are poorly matched, its usage is discouraged [54]. The TTF theory assumes that users are intelligent enough to continue using a technology as long as it adequately meets their needs. User acceptance of this model is based on the understanding that the TTF model consists of three critical components [55]. In various fields, including e-learning technology and metaverse digital library services, the TTF model has been utilized to determine or predict usage intentions [56].

### 2.7 Unified Theory of Acceptance and Use of Technology (UTAUT)

Prior development of the Unified Theory of Acceptance and Use of Technology (UTAUT), there were several theories attempting to explain why people adopt information systems [57]. However, these theories were limited in their ability to fully explain technology adoption as they only covered certain aspects and often overlapped in their explanations. Recognizing the need for a comprehensive framework, researchers emphasized the importance of integrated theories to explain technology acceptance among users. In response to this demand, [58] proposed the UTAUT by combining eight existing theories and models, including the Theory of Reasoned Action, Theory of Planned Behavior, Technology Acceptance Model (TAM) [59], Motivational Model, Model of PC Utilization, Innovation Diffusion Theory, and Social Cognitive Theory.

UTAUT identifies four variables influencing user intention to adopt an information system: performance expectation, effort, social impact, and facilitating conditions [60]. Performance expectancy reflects users' anticipated benefits from technology use [61]. Users' performance expectancy, effort expectancy, social influence, and facilitating conditions collectively determine their perception and adoption of an information system. Performance expectancy reflects the belief that the system will boost work performance, effort expectancy gauges user-friendliness, social influence measures external expectations, and facilitating conditions assess resource availability and support [62].

UTAUT proposes that the relationship between independent variables and behavioral intention is influenced by gender, age, experience, and voluntariness of use, contradicting the initial assumption of a direct influence. Users' perceptions influence their attitude towards the system, which affects their behavioral intentions. Satisfaction with the information system plays a vital role in determining users' behavioral intentions, as satisfied users are more likely to continue using the system and engage in positive word-of-mouth.

The UTAUT theory is widely used and applied in diverse contexts, such as online forums, shopping, banking, e-government, and e-learning. This study serves as the overarching theory to examine user acceptance of the metaverse [63]. To examine the influence of the independent variables in the UTAUT, the study controls for moderating variables such as gender, age, experience, and voluntariness of use [64]. Additionally, the study considers media richness, which motivates user engagement, and information overload, which relates to the difficulty of processing information and its acceptability to consumers.

### 2.8 A unified information theory

Recent studies have shown that a combination of DM ISS models can serve as a supplementary model. Our investigation into the utilization of metaverse technology in digital library services was based on the DM ISS model and other related theories. Individual performance expectancy, as an important factor in user satisfaction within an organizational context, has been identified [65]. Notably, user satisfaction was found to be significantly influenced by individual performance reviews [66]. Several studies have suggested the integration of UTAUT with other models, such as TTF, to enhance the user interface of digital libraries [67].

TAM, for example, has demonstrated a willingness to embrace digital technology. UTAUT has further clarified the acceptance of digital library services in conjunction with ITM [68]. By unifying the TTF model and UTAUT, we can elucidate the mediating relationship between TTF and UI [69]. It has been reported that the TTF model alone is insufficient to describe adoption intention, whereas the combination of TTF and UTAUT yields better results. Therefore, in our study, we integrate UTAUT, DM ISS and TTF models to provide more comprehensive evidence, considering the significance of our findings and their potential impact.

## 3 Research hypotheses

**Figure 1:**
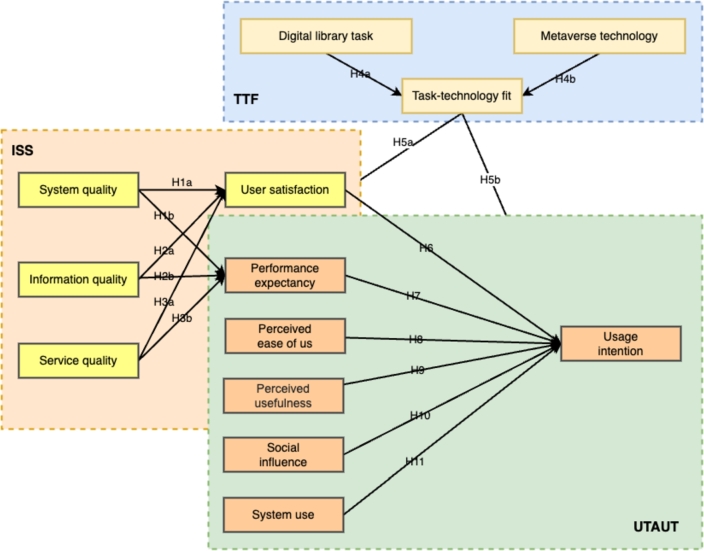
A unified system information model.

In general, the term “continuous usage intention” refers to a user's strong desire to continue utilizing an information system even after the initial use, indicating their intention to use it in the future. Studies indicate that acquiring new users for a metaverse technology in library services is five times more costly compared to retaining existing users [70]. According to [71], it is crucial to protect and nurture users' continuous usage intention, as the initial adoption is a vital step towards the success of an information system [71]. Therefore, it becomes essential to examine users' continuous usage intention for the long-term development of metaverse technology in library services. To address this, a proposed integrated model combines the DM ISS model and the TTF model, aiming to explain the continuous usage intention of metaverse technology among library service users. Fig. 1 illustrates the research model.

The quality of the information in the metaverse technology is essential and can be evaluated based on its timeliness, accuracy, and relevance [72], [73]. Users have high expectations of receiving reliable and up-to-date information from the metaverse. If the information provided is outdated or inaccurate, users may not believe that the metaverse can enhance their productivity [74]. Moreover, users have the ability to compare the information from the metaverse with real-world information. If there are inconsistencies between the two sources, users may doubt the reliability of the metaverse and its ability to support their tasks. Additionally, users desire timely and accurate information from the metaverse [75]. Therefore, poor information quality can negatively impact user intention and satisfaction. In light of these discussions, this study puts forward the following hypotheses:

• $H_{1}$: Quality of information has no direct effect on user perceived usefulness of the metaverse library services.
• $H_{2}$: Quality of information has a direct and positive effect on the system used to acquire information from the metaverse library platform.
  Additionally, we assess the impact of system quality on information processing when interacting with the user [76]. If the system information lacks quality and fails to cater to the user's varied requirements, they are likely to discontinue utilizing metaverse library services. Hence, it is crucial for the system to be intelligent, provide prompt feedback, and offer a user-friendly experience for retrieving information and performing necessary operations. Our hypothesis is as follows:
• $H_{3}$: System quality of the metaverse library has a direct and positive effect on the user's perceived ease of use.
• $H_{4}$: System quality of the metaverse library has positive effect on the perceived usefulness of the system by the users.
  Service quality can serve as an indicator of how reliable and responsive a service is. These two aspects have the potential to influence both user satisfaction and the individual performance of metaverse technology [72], [73]. For instance, metaverse technology, which refers to teleworking platforms that are familiar to only a limited number of people, requires users to invest time in learning how to utilize them. When users encounter difficulties and seek assistance, they have to wait for a response from the user of the metaverse technology. In such situations, users may become impatient and dissatisfied. They might start doubting the ability of metaverse technology to support their task completion and improve their work performance. Conversely, if metaverse technology offers high-quality services, it can enhance user satisfaction and boost their perception of the usefulness of the technology. In simpler terms, when users believe that metaverse technology can deliver favorable services, they will not only be content with the provided services but also view metaverse technology as beneficial and aligned with their task requirements. Building upon the aforementioned discussions, this study proposes the following hypotheses:
• $H_{5}$: Service quality has a direct and positive effect on the perceived ease of use metaverse library system.
• $H_{6}$: Service quality has a positive effect on the perceived ease of usefulness for using the metaverse library system.
  We evaluate the perceived ease of the metaverse library services. Perceived ease of use refers to the retrieval of information from a system with minimal effort by the system user. Users believe that information retrieval is fast if the system is easy to use. If the information retrieval is difficult the system will suffer [77]. We consider the perceived ease of use as the user's level of satisfaction in information retrieval of the desired from the system to fulfill their work. Thus we hypothesize:
• $H_{7}$: Perceived ease of use has a direct and positive effect on user interaction with the metaverse library system.
• $H_{8}$: Perceived ease of use has positive and direct effect on user intention to use metaverse library system.
• $H_{9}$: Perceived usefulness has a positive and direct effect on user intention to use the metaverse library system.
  We evaluate the perceived interaction of the perceived interaction by users. Here we define perceived interaction as the user's perception of using the metaverse library system for teleworking. The willingness by use to share information on the metaverse library platform implies gain of relevant experience and desire to use the system [78]. We hypothesize:
• $H_{10}$: Perceived interaction has a direct and positive effect on user intention to use the metaverse library system.
  System use posits that there is a positive correlation between the ease of use and functionality of a system and its adoption rate among users. It suggests that if a system is user-friendly and effectively meets the needs of its users, it will be more readily accepted and utilized by individuals and organizations. Conversely, if a system is difficult to navigate or lacks essential features, its adoption rate may be lower, and users may be more inclined to seek alternative solutions [79]. We hypothesize:
• $H_{11}$: System use has a direct and positive effect on user intention to use the metaverse library system.

## 4 Materials and methods

### 4.1 Research design and instruments

The primary objective of this study is to investigate the factors that influence the usage intentions of a metaverse technology in the library system using the unified system information theory model. To achieve this, we employ a quantitative approach by using a questionnaire survey (see Table A.7). To develop the questionnaire, we draw upon existing research literature, specifically relying on a reliable and valid questionnaire developed by [80]. All study materials are adapted from previous research but tailored to the specific context of the metaverse library system. This adaptation is crucial to ensure the accuracy and dependability of the gathered information. Each construct within the questionnaire consists of three to four components. In terms of the response format, we employ a Likert scale with five levels, ranging from “strongly disagree” to “strongly agree.” Adjustments were made to certain items, specifically those related to perceived usefulness and ease of use, which were modified based on the recommendations of [81]. A survey instrument was developed to evaluate the model, drawing upon existing published literature. The items and scales related to the constructs of continuous usage intention were adapted from [82]. Regarding the constructs of system quality, information quality, and service quality, the selection of items and scales was based on [82]. The constructs of satisfaction were derived from [83], while the constructs of performance expectancy were informed by the research of [58] for the selection of items and scales [84]. The constructs of perceived interaction were adapted from [85] for the selection of items and scales. Lastly, the items and scales for the constructs of the Task-Technology Fit (TTF) model were adapted from [86]. To ensure the reliability of our findings, we apply the reliability measures established in a previous study [87]. By following this rigorous methodology, we aim to provide valuable insights into the factors that influence the adoption and utilization of metaverse library systems.

### 4.2 Data collection

The study targeted frequent library users that utilize metaverse technology on digital library platform. Recruitment was facilitated using Universitas Terbuka's library resources. The study encompassed frequent library users including university academic staff and students, resulting in the participation of 355 individuals in the lab experiment. However, only 5 individuals were absent due to prior commitments. To ensure data accuracy, five participants were excluded from the analysis as outliers, as they provided false responses (*i.e.* intentionally providing incorrect or misleading responses when completing the questionnaire) on the questionnaire. Thus, a total of 350 reliable data points were collected. The laboratory experiment followed the following procedure to test the hypotheses. Initially, an overview of the metaverse platform's functioning was provided, followed by an explanation. Subsequently, a discussion room was created on the metaverse platform for a 15-minute lecture. Participants then engaged in open discussions regarding how new technology is impacting their daily lives. They subsequently presented their findings to other users within their respective groups. Finally, after an adequate period of utilizing the metaverse platform, participants were requested to complete the questionnaire. The demographic information provided by the respondents is presented in Table 1.

**Table 1:** Demographic information.

	Variable	Number (n)	Percentage (%)
	Male	198	56.57
Gender	Female	152	43.43
	**Total**	350	100.00

	18–25	102	29.13
Age (years)	26–35	237	67.71
	Over 36	11	3.143
	**Total**	350	100.00

	Undergraduate	125	35.71
Education Level	Master's	201	57.43
	PhD	24	6.86
	**Total**	350	100.00

	Professional	86	24.57
	Self-employed	16	4.57
Occupation	Office worker	50	14.29
	Student	188	53.71
	Other	10	2.86
	**Total**	350	100.00

	Every day	315	90.00
	2 to 3 times a week	2	0.57
Frequency of Library use	4 to 6 times a week	33	9.43
	Weekly	0	0.00
	**Total**	350	100.00

	1 to 2 hours	72	20.57
	3 to 5 hours	95	27.14
Time spent in Library (Hours)	5 to 6 hours	128	36.57
	More than 6 hours	55	15.71
	**Total**	350	100.00

	To connect with teachers and administers	89	25.43
	Complete educational tasks	72	20.57
	Seeking information	86	24.57
The motive to use library	To make social connections	48	13.71
	entertainment and playing games	10	2.86
	To connect with other researcher	45	12.86
	**Total**	350	100.00

### 4.3 Data analysis

In this study, we have employed Partial Least Squares Structural Equation Modeling (PLS-SEM) to assess our model and test our hypotheses. The rationale behind adopting the PLS-SEM method is its ability to integrate both path analysis and factor analysis (FA), providing a more effective approach to explore the relationships between multiple variables in a structural equation model, as compared to covariance-based SEM [88]. By utilizing PLS-SEM, we can compare and evaluate various theoretical models while simultaneously handling multiple dependent variables. Our objective with PLS-SEM is to investigate the contextual factors that drive users' intention to use the metaverse technology in library systems [89]. To implement and interpret the results of the PLS-SEM model, we utilized the R package plspm.

## 5 Results

### 5.1 Reliability and validity test

In our investigation, we examined the internal consistency of our measures using Cronbach's alpha [90]. Based on the existing literature, we established that a Cronbach's alpha value above 0.7 indicates a high level of internal consistency. Furthermore, we evaluated the dimensionality of the block using the Dillon-Goldstein (DGrho) value, which we set at DGrho≤0.7. This index is considered more reliable than Cronbach's alpha as it takes into account how well the latent variable explains the block of indicators. Since the DGrho value exceeded 0.7, Table 2 displays the indicators that exhibit a higher level of internal consistency and support homogeneity. The corresponding DGrho values for the two constructs, perceived usefulness and perceived ease of use, are 0.558 and 0.572, respectively, which are relatively low compared to the predetermined limit. To assess convergent validity, we measured the level of variance among the indicators using the confirmatory factor analysis test. Convergent validity is determined based on the extent to which items are correlated with each other [91]. The results of the convergent validity test are presented in Table 2. The instrument demonstrates clear convergent validity with an average extracted variance (AVE) exceeding 0.5 and total factor loadings for each construct surpassing 0.7. An average AVE of 0.5 indicates that the construct explains at least half of the variance in its variables. Analyzing the results further, we found that four factor loadings (INFQ3, SERQ2, SERQ3, SERQ3) fell below the threshold of 0.7, indicating insufficient reliability.

**Table 2:** Standardized loading, reliability, and effectiveness measures of variables.

Construct	Indicators	LF	SL (t-value)	Rd	CR	CA	DG rho	AVE
	INFQ1	0.892	0.796	0.0020				
Information quality (INFQ)	INFQ2	0.679	0.461	0.0115				
	INFQ3	0.567	0.321	0.0080	0.8890	0.8190	0.8350	0.5370
	INFQ4	0.829	0.687	0.0172				

	SYSQ1	0.784	0.615	0.0154				
System quality (SYSQ)	SYSQ2	0.812	0.659	0.0165	0.8850	0.7840	0.7180	0.5020
	SYSQ3	0.802	0.643	0.0161				
	SYSQ4	0.772	0.596	0.0149				

	SERQ1	0.753	0.567	0.0142				
Service quality (SERQ)	SERQ2	0.693	0.480	0.0120	0.8380	0.7570	0.7910	0.5770
	SERQ3	0.791	0.626	0.0156				
	SERQ4	0.862	0.743	0.0186				

	SYSU1	0.711	0.506	0.0126				
System use (SYSU)	SYSU2	0.732	0.536	0.0134	0.8130	0.7860	0.7910	0.5140
	SYSU3	0.729	0.531	0.0133				
	SYSU4	0.798	0.637	0.0159				

	PEOU1	0.719	0.517	0.0129				
Perceived ease of use (PEOU)	PEOU2	0.709	0.503	0.0126	0.8540	0.7890	0.5720	0.5630
	PEOU3	0.569	0.324	0.0081				
	PEOU4	0.761	0.579	0.0145				

	PERU1	0.744	0.554	0.0138				
Perceived usefulness (PERU)	PERU2	0.792	0.627	0.0157				
	PERU3	0.599	0.359	0.0090	0.8490	0.8140	0.5580	0.5290
	PERU4	0.562	0.316	0.0079				

	PERI1	0.842	0.709	0.0177				
Perceived interaction (PERI)	PERI2	0.791	0.626	0.0156	0.8810	0.8870	0.8560	0.6580
	PERI3	0.812	0.659	0.0165				
	PERI4	0.719	0.517	0.0129				

	IU1	0.756	0.572	0.0143				
Intention to use (IU)	IU2	0.692	0.479	0.0120				
	IU3	0.756	0.572	0.0143	0.8650	0.8940	0.6720	0.5670
	IU4	0.791	0.626	0.0156				

	AU1	0.714	0.510	0.0127				
Actual use (AU)	AU2	0.745	0.555	0.0139	0.8360	0.7640	0.7810	0.5770
	AU3	0.732	0.536	0.0134				
	AU4	0.735	0.540	0.0135				

**FL**: Loading Factor, **SL**: Standardized Loading, **Rd**: Redundancy, **CR**: Composite Reliability, **CA**: Cronbach's Alpha, **DG**: DillonGoldstein's, **AVE**: Average Variance Extracted.

However, all other loadings exceeded the 0.7 cutoff value, suggesting satisfactory reliability (see Table 2). Additionally, we used communalities, which are the square root of the loadings, to assess the accuracy of the loadings. Communalities represent the amount of variation accounted for by the latent variables. If loading is higher than 0.5, it indicates that the latent construct explains more than 50% of the indicator's variability (see Table 2). As for redundancy, which indicates the proportion of an endogenous variable's variance attributable to separate latent variables, there is no specific cutoff or general rule, and the results provide insights into this aspect. Table 3 shows the level of distinction between a primary principle and its associated indicators compared to a secondary principle and its associated indicators [92]. In line with [93], the connection between variables within two structures should be lower than the square root of the average variances shared by items within the same structure. The results presented in Table 3 reveal the correlation coefficients between factors and the square root of AVE. Notably, the square root of AVE for each factor surpassed its correlation coefficients with other factors, indicating exceptional discriminant validity.

**Table 3:** Discriminant validity.

	*R*^2^	INFQ	SYSQ	SERQ	SYSU	PEOU	PERU	PERI	IU	AU
Information quality (INFQ)	0.137	0.889								
System quality (SYSQ)	0.502	0.439	0.885							
Service quality (SERQ)	0.277	0.438	0.438	0.866						
System use (SYSU)	0.214	0.245	0.245	0.438	0.828					
Perceived ease of use (PEOU)	0.563	0.554	0.554	0.245	0.438	0.959				
Perceived usefulness (PERU)	0.529	0.449	0.449	0.554	0.245	0.438	0.972			
Perceived interaction (PERI)	0.258	0.381	0.381	0.449	0.554	0.245	0.438	0.917		
Intention to use (IU)	0.567	0.566	0.566	0.381	0.449	0.554	0.245	0.438	0.946	
Actual use (AU)	0.277	0.336	0.336	0.566	0.381	0.449	0.554	0.245	0.438	0.833

$R^{2}$: Root square

Our findings indicate that the $R^{2}$ value of the variability among structures and their respective terms was greater than the correlation coefficients between structures and other structures. This observation fulfills all the criteria for discriminant validity [94]. Furthermore, the diagonal values surpassing the correlation between constructs demonstrate that our measurement tool possesses adequate construct validity.

### 5.2 Structural model assessment

We utilized the methodology presented by [95] to assess the data in two steps. Initially, we examined convergent and discriminant validity. Subsequently, we evaluated the measurement model to analyze the relationships between the theoretical constructs. The entire dataset was employed to investigate the interactive model. The model fit introduced by [96] as an index, along with incremental fit indices listed in Table 4, to assess the adequacy of the measurements and structural modeling. The structural equation model (SEM) was employed to test the ten hypotheses proposed in this study. The parsimonious fit indices, which set extremely high criteria for acceptability, were all surpassed. Thus, all fit indices indicate that the analytical data align well with the composite model, as displayed in Table 4.

**Table 4:** The structural models' fit indicators.

Model Fit Indices	*χ*^2^/*d.f*.	PCFI	RMR	PNFI	PGFI	RFI	NFI	CFI	GFI	IFI	AGFI	RMSEA
Standard of Fit Index	≤3.00	>0.9	>0.9	>0.9	>0.9	>0.9	>0.9	>0.9	>0.9	>0.9	>0.08	>0.08
Measurement Model	1.271	0.921	0.902	0.912	0.940	0.930	0.922	0.986	0.942	0.987	0.034	0.021
Structural Model	1.311	0.916	0.937	0.944	0.941	0.913	0.913	0.986	0.942	0.987	0.031	0.038
Results	Yes	Yes	Yes	Yes	Yes	Yes	Yes	Yes	Yes	Yes	Yes	Yes

$\chi^{2}/d.f$**.**, chi-squared divided by degrees of freedom, **GFI**: goodness-of-fit index, **AGFI**: adjusted goodness-of-fit index, **NFI**: normed fit index, **CFI**: comparative fit index, **IFI**: incremental fit index, **RFI**: relative fit index, **PGFI**: parsimony goodness-of-fit index, **PCFI**: parsimonious comparative fit index, **PNFI**: parsimonious normed fit index, **RMR**: root mean square residual, and **RMSEA**: root mean square error of approximation.

### 5.3 Hypothesis test

Table 5 presents the results showing that three paths (H10; p≤0.05) did not receive support, whereas the remaining paths were statistically significant at the 0.05 level. Table 5, provides detailed information regarding the explanatory paths, such as path coefficients and hypothesis test outcomes for the theoretical model. The findings strongly support the overall unified model. It is evident that the expectation of performance plays a crucial role in influencing the intention to continue using a particular factor. In summary, the analysis confirms the following key points.

**Table 5:** Hypotheses test analysis.

Hypothese	Path	Estimate	S.E.	Path Coefficients	p-value	T-value	Results
H1a	System quality (SERQ)→User satisfaction (US)	0.2351	0.045	2.951	0.0164	4.891	Supported
H1b	System quality (SERQ)→ Performance expectancy (PE)	0.2211	0.051	2.253	0.0214	5.872	Supported
H2a	Information quality (INFQ)→User satisfaction (US)	0.1673	0.039	1.231	0.0423	5.192	Supported
H2b	Information quality (INFQ)→Performance expectancy	0.1212	0.043	1.269	0.0331	5.875	Supported
H3a	Service quality (SERQ)→User satisfaction (US)	0.1314	0.028	1.753	0.0251	2.876	Supported
H3b	Service quality (SERQ)→ Performance expectancy (PE)	0.1214	0.025	1.633	0.0415	3.853	Supported
H4a	Performance expectancy (PE) → User satisfaction (US)	0.2423	0.034	4.144	0.0365	5.014	Supported
Hb	Performance expectancy (PE) → Usage Intention (UI)	0.1132	0.029	2.837	0.0412	3.109	Supported
H5a	Task characteristics (TC) → Task-Technology Fit (TTF)	0.1351	0.021	4.101	0.0492	6.015	Supported
H5b	Tech characteristics (TC) → Task-Technology Fit (TTF)	0.1254	0.034	4.383	0.0426	6.912	Supported
H6a	Task-Technology Fit (TTF) → Performance expectancy (PE)	0.1231	0.046	4.128	0.0372	2.986	Supported
H6b	Task-Technology Fit (TTF)→ Usage Intention (UI)	0.1058	0.053	4.318	0.0211	2.193	Supported
H7	User satisfaction(US) → Usage Intention (UI)	0.2145	0.041	4.144	0.0235	5.318	Supported
H8	Perceived ease of use (PEOU) → Usage Intention (UI)	0.1322	0.036	2.137	0.0341	2.892	Supported
H9	Perceived usefulness (PERU) → Usage Intention (UI)	0.2152	0.124	0.349	0.0277	-2.259	Supported
H10	Social influence (SI) → Usage Intention (UI)	0.1411	0.049	0.392	0.0617	4.326	Not Supported
H11	System use (SYSU) → Usage Intention (UI)	0.2114	0.027	0.352	0.0423	-3.299	Supported

**S.E**: Standard Error

### 5.4 Path coefficients analysis

**Figure 2:**
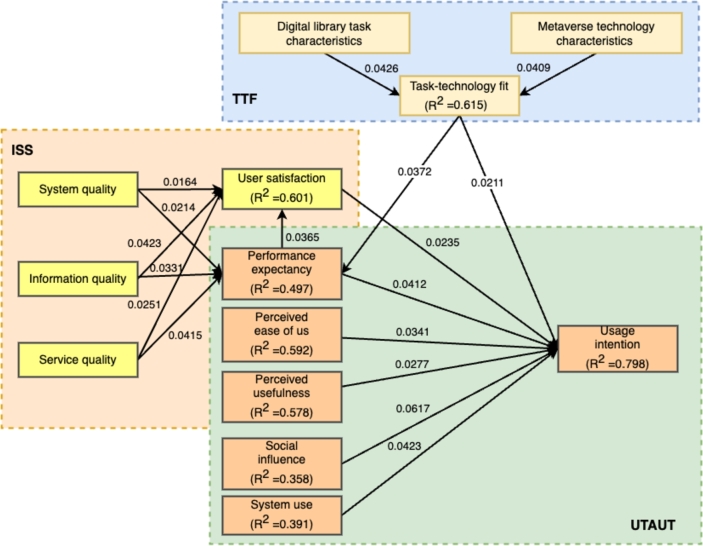
Unified Model path analysis.

In this analysis, we investigate the variations in the intention to use metaverse technology in digital library services among academic staff and students. We compared two user groups, and several key findings emerged. Firstly, academic staff and university students displayed distinct demographic characteristics, including differences in educational level, assignment, and user satisfaction gaps. Secondly, despite having less exposure to metaverse technology and engaging in fewer research activities, student users were found to be the primary adopters of this technology. Therefore, analyzing the divergent usage patterns between academic staff and university students can contribute to a better understanding of user intention (UI). The study identified PEOU, INFQ, SYSQ, SERQ, and AU as immediate determinants of UI and examined their influence, hypothesizing that the roles of academic staff and university students would moderate the impact of these factors on UI. The analysis results from Fig. 2 indicate that the explained variance for the variables of task technology fit, user satisfaction, performance expectancy, perceived ease of use, perceived usefulness, social influence, system use, and usage intention were 0.615, 0.601, 0.497, 0.692, 0.578, 0.358, 0.391, and 0.798, respectively. These suggest that task technology fit, user satisfaction, and usage intention have a strong impact on the overall system use, while perceived ease of use and perceived usefulness also play significant roles. Additionally, performance expectancy and social influence contribute moderately to the variance, highlighting their relevance in the context of the analysis.

In addition, Table 6 shows the empirical research findings regarding the moderating effects of the hypotheses (H1-H11). Initially, the moderating effects of academic staff and student groups were found to be statistically insignificant for both the p-values of SI on UI and AU on UI. However, significant differences in moderation effects between academic staff and student groups were observed for four other p-values. In the student group, PE (β=0.1673,p≤0.0013), SYSU (β=0.1732,p≤0.0029), and US (β=0.1354,p≤0.0025) strongly influenced UI, whereas they had no significant impact in the academic group. In contrast, TTF (β=0.2351,p≤0.0015) significantly affected UI at a 1% statistical significance level in the academic group. Table 6 provides a summary of the differences in path coefficients.

**Table 6:** Path coefficients analysis.

Path	*β*	p-value	T-value
Performance expectancy (PE)→ Usage Intention (UI)	0.1673	0.0013	7.269
Task-Technology Fit (TTF) → Usage Intention (UI)	0.2351	0.0015	6.923
User satisfaction (US)→ Usage Intention (UI)	0.1354	0.0025	4.562
Perceived ease of use (PEOU) → Usage Intention (UI)	0.2443	0.0041	4.234
Perceived usefulness (PERU) → Usage Intention (UI)	0.1452	0.0013	4.321
Social influence (SI)→ Usage Intention (UI)	0.1058	0.0021	5.912
System use (SYSU) → Usage Intention (UI)	0.1732	0.0029	2.898

The study examined both direct and indirect effects to understand the factors that influence the use of services offered by the metaverse library. Indirect effects refer to influences that are mediated by at least one intervening variable, while direct effects are influences that are not mediated by other variables in the model. However, the comparison between direct and total effects revealed that the concept of indirect effects lacked practical significance, indicating that the definition of indirect effects was insufficient [97].

## 6 Discussion

### 6.1 Practical implications

As the usage of digital library services continues to grow with the rapid expansion of metaverse technology, it becomes essential for service providers to address the needs of professionals and scientists to encourage their utilization of these services. In this study, we focused on investigating key factors that impact the intention to use metaverse technology in digital library services. Through an analysis of user experiences in digital library services using the Metaverse platform, we identified several influencing factors, including usability, system quality, service quality, and information quality. These factors were found to significantly impact users' willingness to adopt metaverse technology for accessing digital library services. Our empirical analysis integrated the Task-Technology Fit (TTF) model into the Unified Theory of Acceptance and Use of Technology (UTAUT) and DeLone and McLean's Information Systems Success (ISS) model. This integration allowed us to overcome the limitations of previous studies and provide a more comprehensive understanding of user intention in the context of metaverse technology and digital library services. We found that information and system quality, as well as usability and service quality, play critical roles in user satisfaction and intention to use metaverse technology. Digital library service providers should prioritize these factors to enhance user satisfaction and intention among metaverse users. As indicated in Table 6, we noticed that PE (β=0.1673,p=0.0013), SI (β=0.1068,p=0.0021), US (β=0.1354,p=0.0025), and PEOU (β=0.2443,p=0.0041) greatly influenced UI. Based on the study's findings, service providers should focus on improving information and system quality, as well as user-friendliness and service efficiency. By addressing these factors, digital library services can better meet the perceived ease of use and usefulness for users, ultimately increasing user intention to adopt metaverse technology. Therefore, by understanding the factors influencing user intention, service providers can optimize their offerings and create a more engaging and satisfying experience for metaverse users accessing digital library services.

### 6.2 Theoretical implications

In this study we have integrated different models in the context of digital library services and utilizes metaverse technology and users' experiences as moderating variables. The aim was to establish user intention of metaverse technology in digital library services. Our analysis provides valuable insights to understand the factors that significantly impact user intention which can aid metaverse library technology and other digital library services providers strategies to develop better services for the users. In our study we integrate DM ISS (DeLone and McLean's Information System Success Model), TTF (Task-Technology Fit), and UTAUT (Unified Theory of Acceptance and Use of Technology) to explain user intention in metaverse libraries. The findings indicate that DM and TTF not only directly influence the user interface but also indirectly affect it through user-perceived usefulness, ease of metaverse technology, and information quality. This study makes three significant contributions.

Firstly, the integrated model shows higher predictive power for user acceptance compared to using individual models in isolation. Therefore, future research should adopt an integrated approach when assessing the willingness to use digital library services through metaverse technology systems, as it provides more valuable insights. Secondly, the study addresses the gap in research on long-term adopters of digital library services via metaverse technology, contributing to a limited understanding by examining influencing factors along with cultural aspects. Thirdly, the testing method used in this study improves the effectiveness of evaluating various scenarios and identifying specific deficiencies in research on digital library services. The comprehensive approach presented can serve as a model for evaluating technical factors, while also establishing a baseline for future studies on the impact of system quality. Here, we provide insights on integration of models, user intention in metaverse libraries, long-term adoption of digital library services, and evaluating technical factors. This provides a strong foundation for future academic research and offers guidance for market development strategies in the field of metaverse library technology and digital library services.

### 6.3 Managerial implications

This research highlights the significant role of factors such as DM ISS, TTF, and UTAUT in influencing the willingness of users to utilize digital library services through metaverse technology. It emphasizes the importance of aligning tasks with technology to enhance service quality and emphasizes the need for digital library providers to improve their adaptability to metaverse technology. Different user groups have distinct concerns and needs, and tailored services that align with specific tasks can increase user intention to use metaverse library services. Improving system and information quality, as well as aligning tasks with technology, can enhance user satisfaction and perceived ease of use. Providers should focus on upgrading and enhancing their systems and information to fully leverage the potential of metaverse technology. Outdated or inaccurate contextual information can impact user satisfaction, and providers should prioritize improving the perceived ease of use of metaverse technology in digital library services. User satisfaction is crucial for technological adoption, and incorporating user-friendly features and emphasizing ease of use in promotional campaigns can support various tasks. Policymakers should consider these factors when developing a comprehensive strategy for sustainable growth in digital library services via metaverse technology.

## 7 Conclusion

In this study, we investigated the factors influencing users' intention to use metaverse technology in digital library services. To achieve this, we developed a comprehensive and unified model by integrating the UTAUT, DM ISS, and TTF models. The empirical analysis revealed that system quality, service quality, information quality, perceived ease of use, and perceived interaction significantly influence user satisfaction and intention to adopt metaverse technology in digital library services. Our unified model exhibited greater predictive power compared to individual models, offering valuable insights for future research. We emphasized the importance of considering long-term metaverse technology adopters and cultural aspects in the study of digital library services. The findings provide practical guidance for digital library service providers, highlighting the significance of improving system quality, information quality, and service quality to enhance user experience and satisfaction. However, certain limitations exist in this study, and further research is needed to validate the findings across different digital library platforms and cultural contexts (*i.e.* social sign). Additionally, incorporating other theories and concepts could further enrich our understanding of user behavior concerning metaverse technology. Overall, this study contributes to advancing knowledge in the field of digital library services and provides valuable insights for practitioners to enhance their services. By considering the identified factors and incorporating user feedback, digital library service providers can improve the quality and usability of their metaverse technology, ultimately leading to greater user satisfaction and intention to use.

## Declaration of Competing Interest

The authors declare that they have no known competing financial interests or personal relationships that could have appeared to influence the work reported in this paper.

## Figures and Tables

**Table A.7:** Survey questionnaires, constructs, and measurement items.

Constructs	Measurement Items	Sources
	The system's response time is exceptionally fast.	
System quality	The performance of the Metaverse Technology is consistently reliable.	[48], [98]
	The digital library provided by Metaverse Technology offers a user-friendly experience.	
	Operating the Metaverse Technology is remarkably convenient.	

	The Metaverse Technology surpasses my expectations in terms of service quality.	
Service quality	The overall service quality provided by Metaverse Technology is excellent.	
	I have experience with reliable service from Metaverse Technology.	[48], [98]
	The Metaverse Technology promptly responds to my requests.	

	Metaverse Technology consistently furnishes me with ample information.	
Information quality	Metaverse Technology consistently provides me with precise information.	[48], [98]
	Metaverse Technology consistently provides me with current information.	
	The information provided by Metaverse Technology is consistently dependable.	

	I am highly satisfied with utilizing Metaverse Technology on the digital library platform.	
Satisfaction	I am completely contented with employing Metaverse Technology on the digital library platform.	
	I am genuinely pleased with utilizing Metaverse Technology on the digital library platform.	[99], [100]
	I am extremely happy with utilizing Metaverse Technology on the digital library platform.	

	The utilization of Metaverse Technology allows me to expedite job completion.	
Performance expectancy	Metaverse Technology significantly enhances my productivity.	
	Tasks become more manageable with the aid of Metaverse Technology.	[58], [60], [101]
	Metaverse Technology proves to be invaluable in the context of my professional duties.	

	I will continue utilizing Metaverse Technology in the future.	
Usage intention	I will maintain my frequency of using Metaverse Technology in the future.	
	I intend to use Metaverse Technology over other software in the future.	[102], [103]
	I will gladly recommend Metaverse Technology to others.	

	I need to store a share documents anytime anywhere.	
Task characteristics	I need to publish information anytime anywhere.	[104], [105], [106]
	I need to communicate with colleagues anytime anywhere.	

	Metaverse Technology on digital library platform provides ubiquitous services.	
Technology characteristics	Metaverse Technology on digital library platform provides real-time services	[104], [105], [107]
	Metaverse Technology on a digital library platform provides secure services.	

	The functions of Metaverse Technology are sufficient to assist me in completing my job tasks.	
Task technology fit	Overall, the functions of Metaverse Technology adequately fulfill my job requirements.	[104], [105], [108]
	The functions of Metaverse Technology are suitable for aiding me in accomplishing my job tasks.	
